# Antioxidative Effect of Quetiapine on Acute Ultraviolet-B-Induced Skin and HaCaT Cell Damage

**DOI:** 10.3390/ijms19040953

**Published:** 2018-03-23

**Authors:** Pengcheng Xu, Min Zhang, Xueer Wang, Yuan Yan, Yinghua Chen, Wei Wu, Lu Zhang, Lin Zhang

**Affiliations:** 1 Key Laboratory of Construction and Detection in Tissue Engineering of Guangdong Province, Department of Histology and Embryology, School of Basic Medical Sciences, Southern Medical University, No. 1023-1063 Sha Tai Road, Baiyun District, Guangzhou 510515, China; xpcgb1991@126.com (P.X.); zhangmin507@126.com (M.Z.); wyy85123@126.com (X.W.); yyuanmm@163.com (Y.Y.); mrchch@126.com(Y.C.); dzwuyjun@163.com (W.W.); 2 Key Laboratory of Functional Proteomics of Guangdong Province, Department of Pathophysiology, School of Basic Medical Sciences, Southern Medical University, Guangzhou 510515, China; zlulu70@126.com

**Keywords:** UVB, quetiapine, acute photodamage, p-p38, oxidative stress

## Abstract

Quetiapine is a new type of antipsychotic drug, with effective protection of pheochromocytoma PC12 cells from oxidative stress-induced apoptosis. Ultraviolet-B radiation can increase reactive oxygen species (ROS) production, resulting in significant inflammatory responses in damaged skin. Thus, the purpose of this study is to explore whether quetiapine protects the skin from intermediate-wave ultraviolet (UVB)-induced damage through antioxidant stress. In vivo, we found quetiapine treatment was able to significantly decrease skin thickness, erythema, and edema, as well as inflammation compared to control group. Moreover, quetiapine treatment increased the activities of antioxidant enzymes, including superoxide dismutase (SOD) and glutathione peroxidase (GSH-Px). In addition, it reduced the production of malondialdehyde (MDA), a kind of oxidized lipid. In vitro, we found that quetiapine blocked UVB-induced intracellular ROS generation and maintained the cell activity at a normal level. Furthermore, we tested the phosphorylation of p38 both in vivo and in vitro, and we found that quetiapine could inhibit phosphorylation of p38, which is caused by UVB irradiation. We concluded that quetiapine was able to relieve UVB-induced skin damage through its antioxidative properties. These effects might be associated with p38 MAPK signaling pathway.

## 1. Introduction

Environmental pollution has contributed to the destruction of the atmospheric ozone layer, which has increased ultraviolet (UV) radiation levels. Excessive absorption will cause various degrees of damage, such as erythema solare, photosensitive skin reactions, photoaging of skin, and even DNA damage, and also represents a definitive risk factor for skin cancer [1,2,3]. UV radiation accounts for roughly 13% of sunlight, and it is divided into longwave UV (UVA), intermediate-wave UV (UVB), and shortwave UV (UVC). UVC is completely absorbed by the atmosphere, and only some UVB and UVA radiation reaches the surface of the earth [4]; thereof, UVB damage is often associated with oxidative stress and inflammation. Reactive oxygen species (ROS) refers to a series of active oxidation products directly or indirectly, including superoxide, etc. Chloasma, vitiligo and others that affect beauty are closely related to oxidative stress [5]. Therefore, UVB (between 290 and 320 nm) exerts the greatest effect on people and has been significantly researched in recent years.

In recent years, there has been a lot of evidence that some atypical antipsychotics usually have neuroprotective effects, and the mechanisms of these functions include the upregulation of superoxide dismutase (SOD)-1 [6], inhibition of the proliferation of malignant cells, and reduction of the inflammatory response [7]. Recent studies have shown that quetiapine, a typical antipsychotic drug, can further improve the symptoms of schizophrenia through relieving and sedative effects as a new type of antipsychotic drug, and can effectively relieve the symptoms of cell death in pheochromocytoma (PC12) induced by oxidative stress [6]. Quetiapine improves schizophrenia symptoms, has sedative effects, and can be safely used with only relatively mild adverse events [8]. Additionally, it can also improve psychiatric symptoms and restore neural and cognitive function after cerebral ischemia by increasing antioxidant defenses, scavenging for free radicals, and reducing the inflammation response [9].

Oftentimes, the development of new drugs requires substantial time and money (including the discovery of new sunscreens and natural preservatives); therefore, it is relatively economical and practical to find the other effects of existing drugs. Interestingly, there is very little research on the therapeutic effect of quetiapine in other diseases and injuries. Combined with its anti-inflammatory effects, therefore, this study investigated the potential for quetiapine to prevent UVB-induced oxidative damage in a Kunming mouse model, and explored its potential mechanisms of action.

## 2. Results

### 2.1. Quetiapine Protects Skin from UVB-Induced Damage 

**Figure 1:**
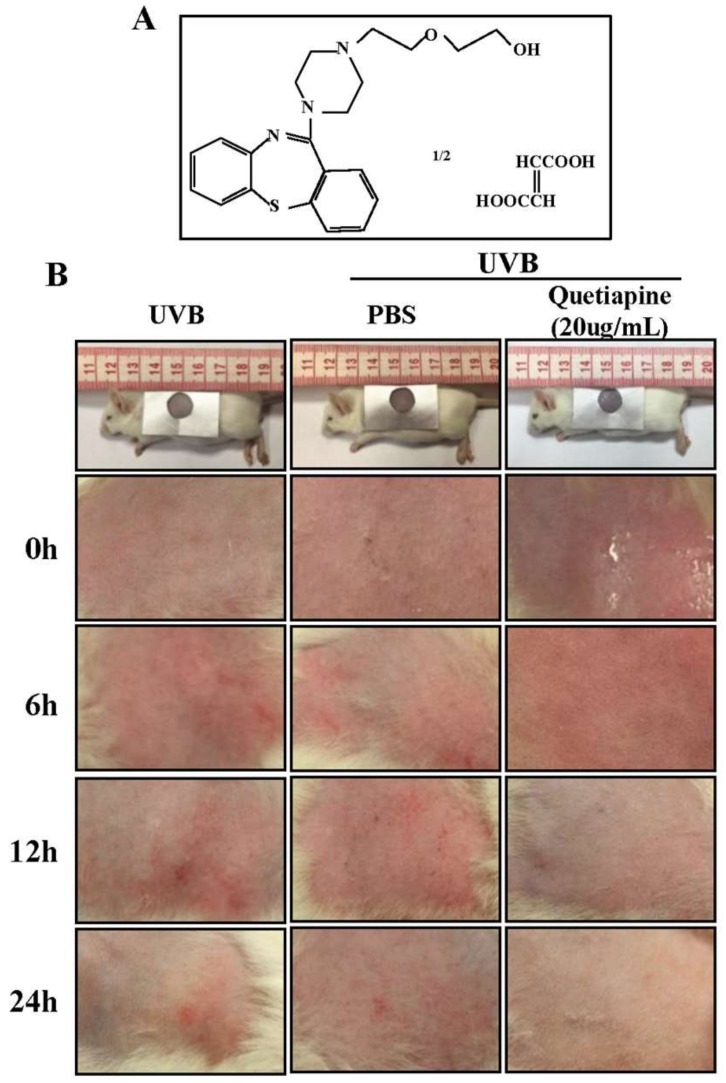
Quetiapine protects skin from UVB-induced damage in vivo. Gross appearance of mouse skin following UV irradiation with or without quetiapine. (**A**) Chemical structure of quetiapine; (**B**) Photographs of mouse skin from different treatment groups at various time points following irradiation (*n* = 5 mice/group/time point).

Firstly, we have verified the protective effect of quetiapine against UV damage in vivo. The results in Figure 1B show that the skin in the quetiapine group formed an oily layer that was absorbed by the skin after approximately 30 min. There was no redness, swelling, or skin folds. Additionally, erythema was absent. Compared to the normal group, quetiapine-treated animals were not grossly different (*n* = 5 mice/group/time point). The skin of the UVB and PBS groups was rougher and had scaling, there was erythema formation, and there was mild congestive inflammation.

**Figure 2:**
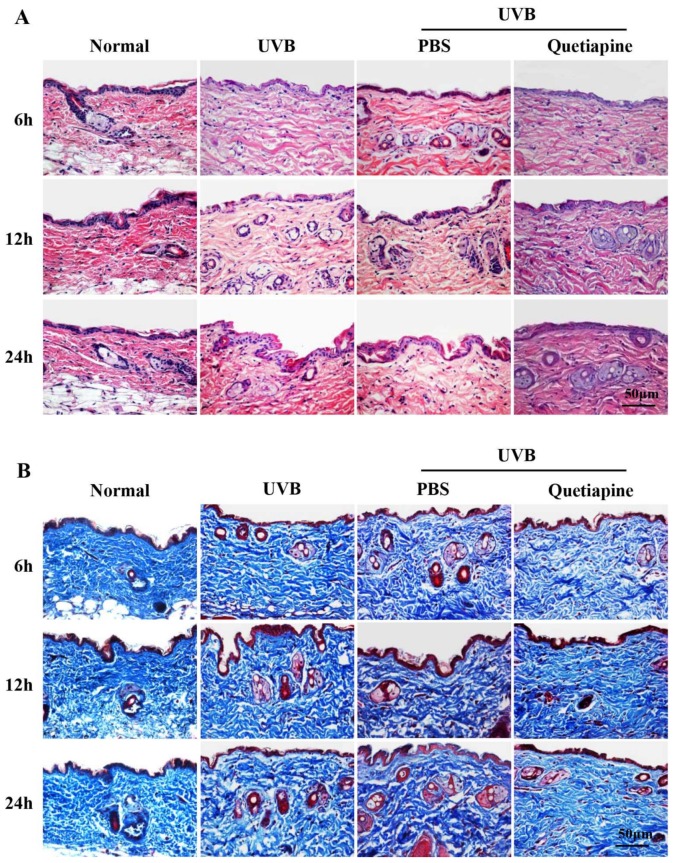
Quetiapine protects skin from UVB-induced damage in histological level. Histological findings after UV irradiation with or without quetiapine protection. (**A**) H&E and (**B**) Masson’s trichrome staining of the different groups at various time points after irradiation. Scale bar = 50 μm (*n* = 5 mice/group/time point).

H&E and Masson’s trichrome staining are shown in Figure 2A,B, respectively. The skin from the quetiapine group was thinner than healthy controls, and the dermis layer had a homogenously distributed fibrous tissue deposition. However, pathological changes were more obvious in the UVB and PBS groups; the epidermal layers had different levels of thickening. Compared to the UVB group, the quetiapine group exhibited less UVB-induced skin damage; the epidermis layer became thin, and the arrangement of fibers in the dermis layer was more ordered.

### 2.2. Effects of Quetiapine on Antioxidants Following UVB Irradiation

**Figure 3:**
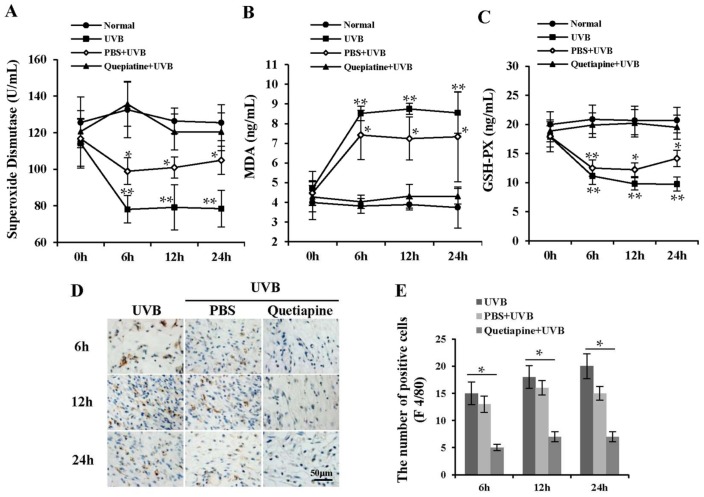
Effects of quetiapine on antioxidants following UVB irradiation reduced the inflammation in vivo. Changes in SOD, GSH-Px, and MDA following irradiation in different groups. (**A**) SOD activity over time; (**B**) GSH-Px activity over time; (**C**) MDA levels over time (*n* = 5 mice/group/time point). (* represents the comparison between UVB group and normal group, * *p* < 0.05, ** *p* < 0.01); (**D**) Representative images of F4/80-postitive cells in skin of the different groups at various time points after irradiation. Scale bar = 50 μm. (**E**) The statistical summary of F4/80 staining was represented in the column chart. (* Represents the comparison between UVB group and quetiapine group).

To further investigate the pathways underlying quetiapine protection against UVB-induced photodamage, the activities of antioxidant enzymes were measured. The results are shown in Figure 3A–C. Compared to the normal group, the simple UVB group had significantly lower SOD and glutathione peroxidase (GSH-Px) activities (*p* < 0.05), and a significantly higher malondialdehyde (MDA) levels (*p* < 0.05). The quetiapine-treated group had significantly lower levels of MDA and significantly higher activities of SOD and GSH-Px compared to the UVB and PBS groups (*p* < 0.05). The levels of SOD, GSH-Px, and MDA were statistically similar to normal controls.

### 2.3. The Treatment of Quetiapine Reduced the Inflammation

UVB radiation often causes an acute inflammatory response; in order to further verify the protective effect of quetiapine, we examined the degree of inflammatory infiltration in different groups. The UVB and PBS groups exhibited obvious immune cell infiltration, and the number of F4/80-positive cells significantly increased compared to the quetiapine groups (Figure 3D). Immune cell infiltration in the PBS group was statistically similar to the UVB group (Figure 3D). The quetiapine group had significantly fewer F4/80-positive cells than the UVB or PBS groups (*p* < 0.05) (Figure 3E).

### 2.4. Quetiapine Increases the Viability of UVB-Treated HaCaT Cells and Protects Cells from ROS-Induced Damage

**Figure 4:**
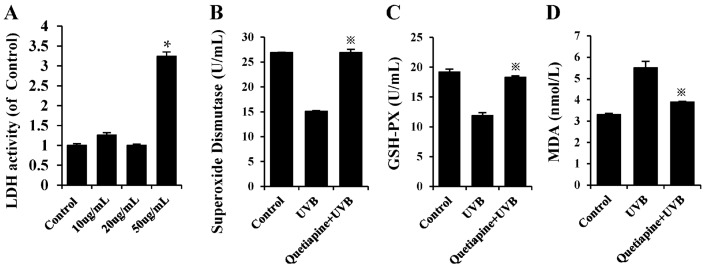
Quetiapine increases the viability of UVB-treated HaCaT cells and protects cells from ROS-induced damage. (**A**) The cytotoxicity assay was determined by measuring LDH activity in the cell culture medium of control, 10, 20 and 50 μg/mL quetiapine group, respectively. ^※^ *p* < 0.05. *n* = 5; (**B**) SOD activity at 24 h after UVB irradiation; (**C**) GSH-Px at 24 h after UVB irradiation; (**D**) MDA levels at 24 h after UVB irradiation; Data are expresses as mean ± S.D., *n* = 5. ^※^ *p* < 0.05. (^※^ Represents the comparison between quetiapine group and UVB group).

UVB radiation also causes cell damage, as well as a series of oxidative stress reactions. To further investigate the effects of quetiapine at the cellular level, as a key target site in acute UVB radiation, HaCaT were cultured for UVB radiation and a series of tests were performed, as shown in Figure 4. As shown in Figure 4A, we evaluated the cytotoxicity of quetiapine by LDH activity assay. The results showed that there was no significant difference among the control, 10 and 20 μg/mL quetiapine groups. While the 50 μg/mL quetiapine shows an obvious cytotoxicity to HaCat cells (*p* < 0.05).

As shown in Figure 4B–D, the activities of SOD and GSH-Px in the quetiapine-treated group were significantly higher than those in the control group (*p* < 0.05), and inversely, the level of MDA was lower than the control group. As a conclusion, we think that quetiapine can reduce damage by increasing antioxidant capacity.

### 2.5. Quetiapine Resists UV Damage through Antioxidant Stress and May Regulate through the p38 MAPK Pathway

**Figure 5:**
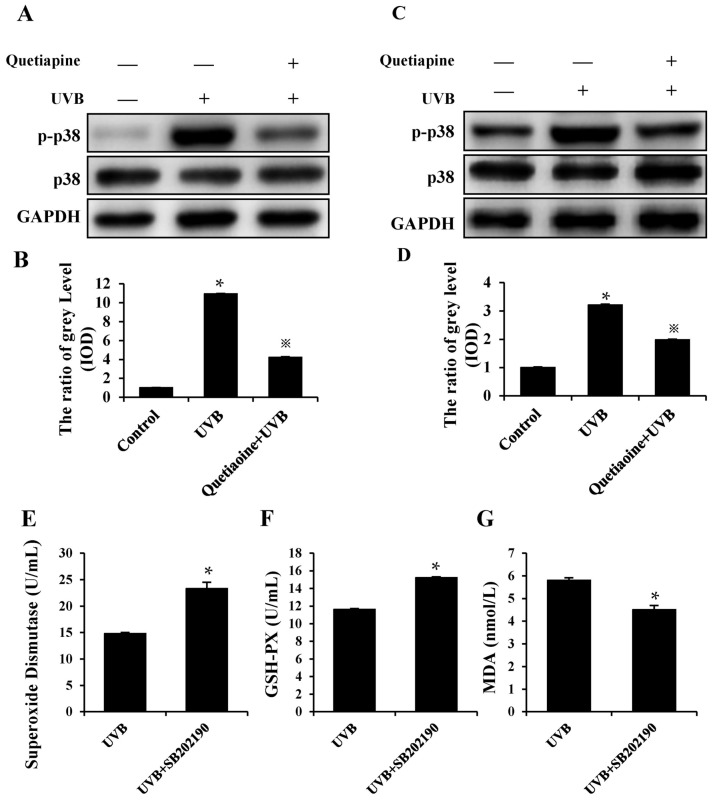
Quetiapine regulates antioxidant stress by the p38 MAPK pathway to resist UV damage. Changes in p-p38 following irradiation in different groups are shown in Figure 5. (**A**) Representative images of p-p38 in the skin of the different groups at the 24 h time point after irradiation, the result of western blot analysis is indicated by the grey level; (**B**) The statistical analysis of the grey level. (* represents the comparison between UVB group and control group, ^※^ represents the comparison between UVB group and quetiapine group, * *p* < 0.05, ^※^ *p* < 0.05); (**C**) Representative images of p-p38 in HaCaT cells of the different groups at the 24 h time point after irradiation; (**D**) The statistical analysis of the grey level. (* represents the comparison between UVB group and control group, ^※^ represents the comparison between UVB group and quetiapine group, * *p* < 0.05, ^※^ *p* < 0.05); (**E**) SOD activity with the activity of p38 inhibited at 24 h after UVB irradiation; (**F**) GSH-Px with the activity of p38 inhibited at 24 h after UVB irradiation; (**G**) MDA levels with the activity of p38 inhibited at 24 h after UVB irradiation; Data are expresses as mean ± S.D., *n* = 5. * *p* < 0.05. (* Represents the comparison between quetiapine group and UVB group).

As is well-known, p38 is a common molecule that is related to oxidative stress. In order to explore one of its possible mechanisms, in combination with its inflammatory response and the antioxidative effect, we examined the phosphorylation level of p38. Our results showed that a difference of p-p38 was present at the protein phosphorylation level between the UVB and quetiapine groups in vivo and in vitro (Figure 5A,C). On the other hand, based on the previous results, we have added the p38 inhibitor (SB202190) to HaCaT cells and irradiated the cells using UVB lamps, as mentioned previously. Then, we detected the secretion of SOD, GSH-Px and MDA; the results showed that the levels of SOD, GSH-Px and MDA were close to or had returned to normal levels (Figure 5E–G). Therefore, we believe that quetiapine resistance UV damage may be regulated by the p38 MAPK pathway.

## 3. Discussion

Substantially greater levels of UVB light reach the earth’s surface than UVA or UVC light [2]. After absorption by skin, UV light generates reactive oxygen species (ROS) and induces oxidative damage to lipids, proteins, and nucleic acids [1,10]. UV damage results in inflammation that can lead to erythema and a skin-scaling phenomenon. In addition, tissue damage caused by acute UVB is due to the apoptosis, inflammation and oxidative damage of keratinocytes, leading to epidermis damage and the loss of barrier function [11,12]. In recent years, different types of drugs (including traditional Chinese medicines and plant extracts) have been investigated for the treatment and prevention of skin photoaging with some success [13,14,15,16,17,18]. The HaCaT cell photodamage model confirmed that UVB radiation can cause cell nucleus and mitochondrial DNA damage. UVB inhibits the proliferation of HaCaT cells during the process of the cell model, resulting in cell apoptosis, which is one of the causes of skin aging. The main mechanism by which long-term UV exposure causes photoaging of the skin is activation of the relevant membrane receptors in the epidermal HaCaT cells, initiating intracellular signaling, resulting in a series of molecular changes including oxidative stress and oxidative damage. At present, the regulation of reactive oxygen species (ROS) in light damage and light aging has been clarified. The increase of reactive oxygen species caused by photoaging can attack the antioxidant defense system that damages the body and lead to the impairment of cell structure.

This study reports a novel protective function for quetiapine against skin photodamage. While quetiapine is canonically a psychiatric drug, this study was implemented based on the previously outlined characteristics and the neuro sedative function of quetiapine. To minimize the psychiatric effects of the drug, this study used the minimum maintenance dose, which is based on the previous studies [19,20]. We utilized an acute UVB photodamage model and showed that UVB irradiation produced redness, swelling, mild erythema, and roughening of the skin surface. Compared to the UVB group or the group given a sham PBS treatment, the quetiapine-treated group exhibited little photodamage. Gross observations and histological observations revealed relatively normal skin when treated with quetiapine before irradiation.

Because quetiapine was absorbed by the skin, its protective function likely did not result from forming a simple barrier. Its effects were due to antioxidant functions following UVB-induced oxidative damage. UV radiation damages cells and tissues in part by reducing the activities of antioxidant enzymes such as SOD [21] and GSH-Px decrease. SOD converts two superoxide anions into H_2_O_2_. H_2_O_2_ is cleared by GSH-Px to protect the cell structure and function. UV radiation exposure to skin also produces more free radicals, and coupled with reduced activities of SOD and GSH-Px, the cellular oxidative balance becomes disrupted. MDA is an end-product of lipid peroxidation, and it can induce cross-linking of membrane proteins and causes photodamage. Our experimental results indicated that quetiapine preserves GSH-Px and SOD activities and protects against the formation of MDA, ultimately reducing the oxidative insult to skin tissue. On the other hand, F4/80-positive immune cells, which are consistent with inflammation, were relatively few in the quetiapine-treated group.

**Figure 6:**
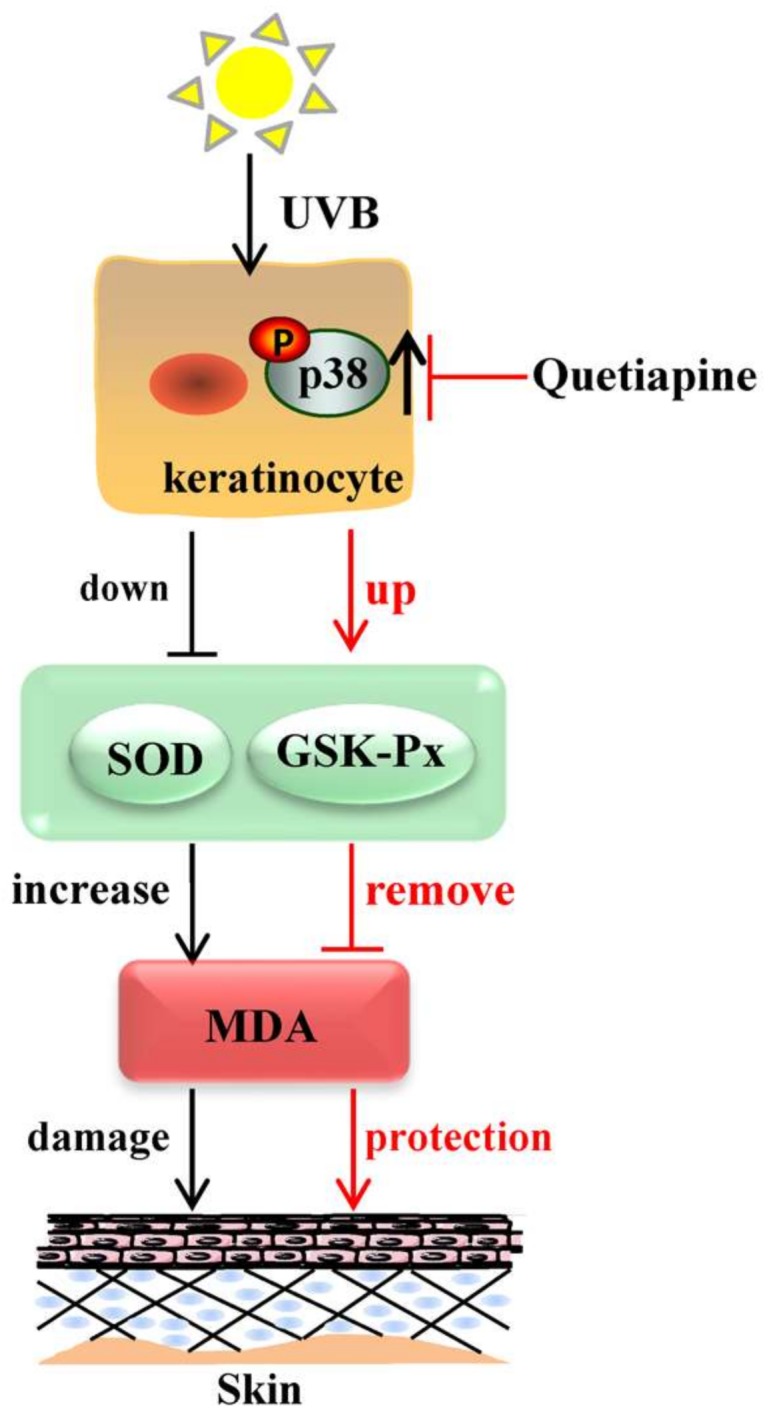
Schematic diagram of the protective response of quetiapine. UVB irradiation can change the expression levels of SOD, GSH-Px and MDA, as well as potentially being regulated through the p38 MAPK signal pathway, and may then cause a range of damage (inflammation injury, apoptosis and so on). The protective mechanism of quetiapine may be regulated by the same pathway, as well.

In the meantime, the results showed that UVB radiation reduces the activity of major antioxidant enzymes (SOD, GSH-Px) in HaCaT cells, decreases the antioxidant capacity, and increases MDA. The quetiapine intervention can alleviate the effect of UVB on the anti-oxidative enzyme activity of human HaCaT cells, leading to an increase in the major antioxidant enzymes (SOD, GSH-Px) to near or even slightly above the normal levels. Quetiapine decreases the MDA level induced by UVB, as well as regulating UVB-induced oxidative stress in human HaCaT cells (Figure 6); it can relieve or even reverse the damage of oxidative stress, and exerts an anti-damage protective effect on cells.

Mitogen-activated protein kinase (MAPK) is an important biological macromolecule whose function is to regulate cell growth and differentiation [22]. A large number of studies have confirmed that the MAPK pathway plays an important biological role in HaCaT cells [23], The research has shown that the MAPK pathway is involved in the regulation of cellular inflammation and apoptosis caused by UVB [24]. For example, some studies have shown that the ERK1/2 signal is involved in the migration of HaCaT cells [25], and some believe that JNK can mediate the apoptosis of HaCaT cells [26]. In addition, studies have shown that p38 MAPK is related to the EGFR internalization of HaCaT cells induced by stress [27]. Although there have been studies on the biological effects of MAPK signals in HaCaT cells, the study of photo biology has not been comprehensive or deep. On the other hand, the secretion of apoptosis and inflammatory factors are early molecular events of HaCaT cells after exposure to UVB, and p38 is important to the early process in HaCaT cells [28]. Therefore, we investigated and found that the level of p38 phosphorylation was increased, and in a rescue assay, it was reversed, proving that p38 has a major role in this process (Figure 6). Therefore, we think that p38 may be a relevant signaling molecule, but the ultraviolet exposure of human skin is more complex.

Our study demonstrates that quetiapine has the ability to prevent the cell and skin from ROS-induced and inflammation-associated damage, and the ROS clearing ability of quetiapine provides protection against UVB irradiation, which may be regulated by blocking p38-MAPK activation, although the signaling pathway needs further exploration. Therefore, we suggest that quetiapine is an effective therapeutic candidate for preventing skin photodamage, but requires further evaluation as a protectant against UV radiation.

## 4. Materials and Methods

### 4.1. Animals

A total of 60 female Kunming mice (SCXK 2011-00115-0107239, 6 June 2015, provided by the Experimental Animal Center of Southern Medical University, Guangzhou, China) between 6 and 8 weeks old, weighing 25–30 g were used. Animals were housed at constant temperature (22–25 °C) and humidity with free access to food and water. Animals were subjected to a 12 h light/dark cycle. All experimental procedures were in compliance with the National Institutes of Health guidelines for Care and Use of Laboratory Animals and were approved by the Bioethics Committee of Southern Medical University.

### 4.2. Quetiapine

Quetiapine was dissolved in Dimethyl sulfoxide (DMSO) and diluted in phosphate-buffered salt solution (PBS). In vivo, the working concentration of quetiapine was the minimum maintenance dose (400 mg/mL) used in clinics, as well as in vitro, it was divided into three concentrations: Group 1 (10 μg/mL); Group 2 (20 μg/mL); Group 3 (50 μg/mL). Based on the CCK-8, the working concentration was 20 μg/mL.

### 4.3. UVB Irradiation

Mice were randomly divided into 4 groups (*n* = 15 per group) that represented treatments or controls. These were: normal healthy controls (no UVB or treatment), UVB irradiation alone (no treatment), UVB irradiation following a sham phosphate buffered saline (PBS) treatment (PBS group), and UVB following quetiapine treatment (quetiapine group). Each of these 4 groups was sub-divided randomly into 4 subgroups (*n* = 3 each), which represented various time points to be investigated. Before experimentation, mice were anesthetized by intraperitoneal injection of 1% sodium pentobarbital and hair was removed from the back skin of mice using hair removal wax.

Parallel UVB lamps (PL-XL 40W, PHILIPS, emission peak at 313 nm) were measured using a UV spectral radiometer to select a UVB (290–320 nm) spectral radiance of 100 J/m^2^ [29]. Mice were placed 10 cm below the lamps, the back skin of mice was collected at 0, 6, 12 and 24 h and divided into two parts. One part was fixed in 4% paraformaldehyde for histology, one part was used for western blot, and the other was frozen at −80 °C for molecular measurements. Mice were photographed during experiments to monitor skin surface changes.

### 4.4. Cell Culture and UVB Irradiation

Human skin keratinocytes, HaCaT, were cultured in DMEM (Gibco BRL, Grand Island, NY, USA) supplemented with 10% heat-inactivated fetal bovine serum (FBS; Gibco, Grand Island, NY, USA) at 37 °C in 5% CO_2_. The cells were incubated with or without quetiapine for 12 h prior to UVB irradiation. Then, the cells were washed with phosphate buffered saline (PBS; pH 7.4) and irradiated using a UVB lamps. The irradiation intensity was monitored by a UVB radiometer (LX-9626, LANDTEK, Guangzhou, China). Immediately after UVB irradiation, the cells were returned to the incubator and incubated with drug-free medium for 24 h.

### 4.5. Cytotoxicity Assay

The cytotoxicity of quetiapine was detected by Cytotoxicity LDH Assay Kit (CK12, DongRen, Shanghai, China) following the manufacturer’s instructions. The cells were seeded in 96-well plates at a concentration of 2000 cells/mL. The cells underwent pretreatment with quetiapine mixed with dimethyl sulfoxide (DMSO) at various concentrations (10, 20 and 50 μg/mL). Before irradiation, the cells were incubated for 24 h and then, after irradiation, the cells were incubated for 24 h. Following this, LDH release agent was added, and the cells were incubated for another 30 min, and then 50 μL of stop solution was added to each sample well and mixed by gentle tapping. Finally, the plates were read immediately on a plate reader at a test wavelength of 490 nm.

### 4.6. Measurement of SOD, GSH-Px, and MDA Levels on HaCaT Cells

The ELISA kits to evaluate SOD (Cat No. AD12043Hu), GSH-Px (Cat No. AD12650Hu) and MDA (Cat No. AD11335Hu) in cell level were bought from AndyGene Biotechnology Co. (Beijing, China). After the cells had undergone the quetiapine treatments, the UVB was applied, with an incubation time after irradiation of 24 h. Then, cell supernatants were harvested and used for detection. The concentrations of SOD, GSH-Px and MDA in the supernatant were determined by ELISA. The absorbance was determined using a microplate reader at a wavelength of 450 nm.

### 4.7. Hematoxylin and Eosin (H&E) and Masson’s Trichrome Staining

The fixed skin samples were embedded in paraffin, sectioned at 4–5 μm and mounted on slides. The sections were deparaffinized, rehydrated in an ethanol gradient, and stained with H&E or Masson’s trichrome according to standard protocols using commercially available reagents.

### 4.8. F4/80 Immunohistochemistry

Following paraffin removal and rehydration, after heat repair and inactivation of endogenous peroxidase, they were blocked at room temperature for 1 h. The primary antibodies for F4/80 (eBioscience, 1:500 dilution) was incubated 4 °C overnight. Biotinylated secondary antibodies (horseradish peroxidase (HRP)-labeled IgG (H + L), Wuhan Boster Biological Technology, Ltd., Wuhan, China) was incubated at room temperature for 30 min. Finally, 3,3-diaminobenzidine (DAB) was added to react with HRP and cell nuclei were counterstained with hematoxylin for microscopy.

### 4.9. Measurement of SOD, GSH-Px, and MDA Levels on Skin

Frozen skin was thawed and was homogenized in normal saline and centrifuged at 4 °C and 10,000 rpm for 10 min. Supernatants were collected and all kits were performed according to manufacturer protocols. SOD activity was determined using a kit (GuChen, Shanghai, China). The kits to evaluate GSH-Px (Cat No. DL-GPX-Mu) and MDA (Cat No. DL-MDA-Ge) were from Dldevelop (Wuxi, China).

### 4.10. Western Blot

The cells were harvested and used to prepare total protein lysates. The skin samples were washed with PBS to remove the blood components, then the skin samples were placed in RIPA, and the protein was harvested by liquid nitrogen. Each sample was resolved via 5% SDS-PAGE and transferred to polyvinylidene fluoride membranes (Millipore, Bedford, MA, USA). The blots were blocked with blocking liquid (Beyotime, Shanghai, China) and incubated with a primary antibody (p-p38, p-38; Cell Signaling, Danvers, MA, USA) at 4 °C overnight, followed by incubation with a secondary antibody, and antibody binding was detected using an enhanced chemiluminescence kit (Millipore, Bedford, MA, USA). The bands were quantified using Gel-Pro software (Media Cybernetics, Rockville, MD, USA) by measuring the band intensity.

### 4.11. Statistical Analyses 

SPSS software (v. 20.0, SPSS, Chicago, IL, USA) was used for analysis. Data are presented as mean ± standard deviation and all groups were analyzed using independent-sample *t* test. *p* Values < 0.05 indicated statistical significance. *p* < 0.01 indicated a significant difference.

## Abbreviations

UVB	Ultraviolet-B
ROS	Reactive oxygen species
SOD	Superoxide dismutase
GSH-Px	Glutathione peroxidase
MDA	Malondialdehyde
PC12	Pheochromocytoma
